# Association of Primary and Specialty Care Integration on Physician Communication and Cancer Screening in Safety-Net Clinics

**DOI:** 10.5888/pcd17.200025

**Published:** 2020-10-29

**Authors:** Ashley M. Kranz, Jamie Ryan, Ammarah Mahmud, Claude Messan Setodji, Cheryl L. Damberg, Justin W. Timbie

**Affiliations:** 1 RAND, Arlington, Virginia; 2 Pardee RAND Graduate School, Santa Monica, California; 3 RAND, Pittsburgh, Pennsylvania; 4 RAND, Santa Monica, California

## Abstract

**Introduction:**

Primary care providers who lack reliable referral relationships with specialists may be less likely than those who do have such relationships to conduct cancer screenings. Community health centers (CHCs), which provide primary care to disadvantaged populations, have historically reported difficulty accessing specialty care for their patients. This study aimed to describe strategies CHCs use to integrate care with specialists and examine whether more strongly integrated CHCs have higher rates of screening for colorectal and cervical cancers and report better communication with specialists.

**Methods:**

Using a 2017 survey of CHCs in 12 states and the District of Columbia and administrative data, we estimated the association between a composite measure of CHC/specialist integration and 1) colorectal and cervical cancer screening rates, and 2) 4 measures of CHC/specialist communication using multivariate regression models.

**Results:**

Integration strategies commonly reported by CHCs included having specialists deliver care on-site (80%) and establishing referral agreements with specialists (70%). CHCs that were most integrated with specialists had 5.6 and 6.8 percentage-point higher colorectal and cervical cancer screening rates, respectively, than the least integrated CHCs (*P* < .05). They also had significantly higher rates of knowing that specialist visits happened (67% vs 42%), knowing visit outcomes (65% vs 42%), receiving information after visits (47% vs 21%), and timely receipt of information (44% vs 27%).

**Conclusion:**

CHCs use various strategies to integrate primary and specialty care. Efforts to promote CHC/specialist integration may help increase rates of cancer screening.

**Summary**

**What is already known on this topic?**

Community health centers (CHCs) provide primary care to disadvantaged populations and have lower-than-average cancer screening rates. Stronger integration of CHCs and specialists is recommended to increase cancer screening, but the impact of integration efforts in the real world is unknown.

**What is added by this report?**

CHCs that are more strongly integrated with specialists have higher rates of cervical and colorectal cancer screening and better communication with specialists compared with the least integrated CHCs.

**What are the implications for public health practice?**

Integration between CHCs and specialists may enhance communication across health care providers and improve cancer screening rates. Efforts are needed to promote integration and identify the mechanisms that lead to long-term, effective partnerships.

## Introduction

Community health centers (CHCs), a type of federally qualified health center, provide primary care to racially/ethnically diverse and economically disadvantaged populations, regardless of ability to pay (1). Given the widespread inequities in cancer screening for racial/ethnic minority populations and the uninsured (2,3), CHCs are well-positioned to close gaps in cancer screening because of their focus on serving vulnerable populations and commitment to quality measurement and quality improvement activities.

Systematic reviews have documented barriers to and strategies for increasing cancer screening (4,5), including those specific to CHCs (6,7). CHC strategies to increase cancer screening include using patient navigators to help patients understand the screening process and complete follow-up appointments (4,6), reminding providers about the importance of screenings (5), giving providers feedback, and tracking the return of tests (5,7). Despite these efforts, colorectal and cervical cancer screening rates in CHCs remain low (1).

Although many cancer screening tests can be conducted in CHCs, specialty care is needed for some tests (eg, colonoscopies) and for follow-up care after patients receive abnormal or positive test results (eg, referrals to gynecologic oncologists for diagnoses of invasive cervical cancer). CHCs have reported that a barrier to cancer screening is their inability to refer patients needing follow-up care (7); thus, stronger integration of specialists and CHCs is recommended to increase screening rates (8). In addition to encouraging screening at CHCs, integration can facilitate communication between CHCs and specialists, which is needed for tracking referrals to specialists and the results of those referrals and coordinating care across multiple providers and settings (8). For example, some CHCs have reported challenges in tracking follow-up care for patients with positive cancer screening results who they referred to specialists (9). Although most CHCs report the ability to exchange information with hospitals and specialists (1), this information may be limited or delayed (10), which may hinder communication between CHCs and specialists and appropriate follow-up care.

Despite the potential benefits of CHC/specialist integration, to our knowledge, no studies have examined its effect on cancer screening rates and CHC/specialist communication. Our study addresses gaps in the literature by combining survey and administrative data to describe the strategies CHCs are using to integrate care with specialists and to examine the extent to which more strongly integrated CHCs have higher rates of screening for colorectal and cervical cancers and report better communication with specialists.

## Methods

### Data sources

This cross-sectional study used a survey developed by the authors and completed in summer 2017 by medical directors at CHCs funded by the Health Resources and Services Administration (HRSA) in 12 states (California, Colorado, Illinois, Louisiana, Maine, Minnesota, New Jersey, Oregon, Utah, Vermont, Washington, and Wisconsin) and the District of Columbia. The survey was fielded as part of a larger effort to describe the landscape of care integration activities involving CHCs, specialty practices, hospitals, and social service organizations (10–12). We purposely sampled states to include those with active Medicaid accountable care organizations (ACOs) at the time of the survey (7 states), because these programs may promote greater accountability for providing more coordinated care across settings. We added 5 states (California, Illinois, Louisiana, Washington, and Wisconsin) and the District of Columbia to improve geographic diversity and to include states that expanded Medicaid under the Affordable Care Act and states that did not. Medicaid expansion may reduce the number of uninsured in a state and ease some barriers to referrals and care coordination. We supplemented these survey data with data from HRSA’s Uniform Data System (UDS) database (13), which provided contact information for medical directors and information on CHC characteristics and quality of care measures.

### Conceptual model

Building on previous research examining strategies to increase cervical and colorectal cancer screening in CHCs (7,8,14), we hypothesized that stronger CHC/specialist integration would increase cancer screening through 2 related mechanisms. First, improving links between CHCs and specialists is a recommended strategy for increasing access to more invasive screening modalities not traditionally offered at CHCs (eg, colonoscopies) (8). Second, because the inability of health care providers to refer patients needing follow-up care is a barrier to cancer screening (7), we hypothesized that more strongly integrated CHCs would conduct more screenings because they have more reliable (or any) referral options available to patients who need additional testing and follow-up care after receiving abnormal screening results. We further hypothesized that stronger CHC/specialist integration would improve communication between CHCs and specialists. Relationships with specialists have been identified as a key component of successful screening programs (8), whereas problems coordinating with external laboratories and specialists have been identified as a barrier to cancer screening (14). Thus, stronger CHC/specialist integration may lead to the establishment of communication agreements and encourage more timely information sharing and better follow-up care. Our study tested the associations between CHC/specialist integration and 1) screening rates for colorectal and cervical cancers and 2) communication between CHCs and specialty care providers.

### Survey

Our main data source was a 30-minute web-based survey completed by CHC medical directors in 12 states and the District of Columbia. The survey gathered information about how CHCs collaborate with hospitals (10), social service organizations (11), and specialists (12), with specialists being the focus of this study. Survey items were informed by a review of the literature and by interviews with subject matter experts and representatives of CHCs and state primary care associations, which provide training and technical assistance to safety-net providers. We used items from existing surveys when possible (15–17). We obtained comments on the draft survey from primary care associations. We conducted cognitive interviews with 3 CHC medical directors outside our sample to ensure that survey items elicited the intended information. We offered respondents an incentive of a $50 gift card for survey completion, which was later increased to $100 to improve the response rate after 4 weeks in the field. Of 407 CHCs invited to complete the survey, 215 responded to the items used in this analysis (response rate, 52.8%).

### Variables

The survey consisted of 12 items (Appendix). We use 4 items related to CHC/specialist communication as dependent variables to indicate whether the CHC “often” or “always” 1) knew that a specialist visit happened, 2) knew the visit’s outcome, 3) received clear recommendations on follow-up and care management after the specialist visit, and 4) received results or recommendations from the specialist in a timely manner.

We constructed a composite measure of CHC/specialist integration to summarize the breadth of strategies used by CHCs to achieve greater integration with specialty care providers. The composite included strategies focused on improving referrals (establishing referral agreements, participating in e-consults, making specialist appointments for patients, and reminding patients about those appointments), aligning goals with specialists (through quality improvement projects or health promotion initiatives), information exchange (ability to send data electronically to specialist, ability to read specialists’ electronic health records [EHRs] in real time), and improving access (by providing specialty care on-site, expanding telemedicine, and developing affiliations with hospitals). An additional variable included in the composite measure provided a count of the types of provisions included in referral agreements between CHCs and specialists. We developed the composite of CHCs’ overall integration with specialists using a 1-factor confirmatory factor analysis. We assessed goodness-of-fit by the comparative fit index (0.78) and the root mean square error of approximation (0.13). Higher values of the integration composite indicate stronger CHC/specialist integration. We categorized CHCs as having low, medium, and high levels of integration by dividing CHCs into tertiles based on the composite measure score. Additional survey items provided information on the characteristics of the survey respondents and characteristics of the CHCs, such as whether the CHC reported “often” or “always” having a chaotic practice environment and having levels of physician turnover that affected the CHC’s ability to care for patients (18). We used the 2011–2015 American Community Survey’s 5-year pooled files to derive a 6-item composite measure of neighborhood socioeconomic status for the primary care service area in which a plurality of each health center’s patient population lives (19).

HRSA’s UDS provides information on rates of screening for colorectal and cervical cancer in CHCs, which includes screenings conducted both at the CHC and off-site (eg, through referral) (13). We also used 2 performance measures from the UDS for falsification tests; we hypothesized that measures of screening for healthy weight among adults and screening for tobacco use (and provision of cessation intervention for active smokers) among adults would be unaffected by CHC/specialist integration. We also obtained data on the following characteristics of patients and CHCs from the UDS: total number of patients; number of service sites; percentage of patients who were uninsured, enrolled in Medicaid, or belonged to a racial/ethnic minority group; number of primary care providers per 10,000 patients; number of enabling-service providers per 10,000 patients; and rural or urban location. All UDS variables are reported at the level of the CHC grantee (and the survey was administered at the grantee level), which often includes multiple service sites (13).

### Analysis

To assess the representativeness of our study sample, we used measures from the 2017 UDS to compare characteristics of CHCs that responded to the survey and those that did not in our surveyed states. Then, we described strategies used by CHCs to integrate care with specialists. Next, we fit linear regression models to examine the association between the composite measure of CHC/specialist integration and rates of screening for colorectal and cervical cancers and to conduct falsification tests using the other UDS measures that were not expected to be associated with CHC/specialist integration. We also use logistic regression models to examine the associations between overall CHC/specialist integration and the 4 measures of CHC/specialist communication. We tabulated the results from the logistic regression models as predicted probabilities for each level of the integration index, leaving all other variables at their actual values, and compared these probabilities across integration tertiles using Wald χ^2^ tests. All regression models adjusted for the aforementioned characteristics and included robust standard errors. We performed all analyses using Stata version 16 (StataCorp LLC) and used *P* < .05 to identify significance. The study was approved by the RAND institutional review board.

## Results

The 215 responding CHCs serve an average of 24,398 patients annually (median, 13,700), primarily in urban communities (Table 1). Across CHCs, one-fifth (20.4%) of patients were uninsured, more than half (53.2%) were enrolled in Medicaid, and 60.2% were racial/ethnic minority patients. CHCs report an average of 9.7 primary care physician FTEs per 10,000 patients, and 40.6% of respondents reported that physician turnover affects the quality of care in their organization. Approximately 1 in 6 respondents (17.9%) report a chaotic practice environment in their CHC. Our sample of CHCs served patients in vulnerable communities, where the annual household income was below the federal poverty level in 20.0% of household incomes, on average. On average, CHCs reported screening 43.1% of eligible patients for colorectal cancer and 54.5% of eligible patients for cervical cancer. On average, CHCs in the highest tertile served more patients, served a higher percentage of patients enrolled in Medicaid, and reported a less chaotic practice environment. Most survey respondents (64.8%) were medical directors and 51.6% had worked at their CHC for more than 5 years. CHCs responding to the survey were similar to nonrespondents on nearly all characteristics, except that responding CHCs served a slightly lower percentage of patients identified as racial/ethnic minority (60.2% vs 66.6%).

**Table 1:** Characteristics of Respondents (N = 215) to a Survey of Community Health Centers (CHCs) in 12 States and the District of Columbia, 2017^a^

Characteristic	All CHCs	By Tertile of Integration^b^		
		Lowest	Middle	Highest
**Cancer screening rate, %**				
Colorectal cancer	43.1	39.8	44.8	44.6
Cervical cancer	54.5	49.2	57.7	56.4
**CHC characteristics**				
Mean no. (SD) of patients	24,398 (29,305)	20,867 (29,672)	21,601 (21,484)	30,046 (34,576)
Mean no. (SD) of service sites	8.4 (8.7)	7.7 (9.1)	7.9 (6.4)	9.5 (10.1)
Racial/ethnic minority patients, %	60.2	55.2	62.4	62.9
Uninsured patients, %	20.4	20.4	21.6	19.1
Enrolled in Medicaid, %	53.2	50.0	53.0	56.6
Mean primary care FTE per 10,000 patients	9.7	9.6	10.0	9.5
Mean enabling-service FTE per 10,000 patients	11.2	12.1	9.3	12.2
CHC has a chaotic environment, %	17.9	23.9	19.1	10.9
Physician turnover affects quality of care, %	40.6	42.8	39.2	39.7
**Regional characteristics**				
Rural, %	38.1	40.8	39.4	34.2
State-expanded Medicaid, %	89.8	88.7	84.5	95.9
State has Medicaid ACO, %	31.6	33.8	35.2	26.0
Composite measure^c^ of SES in CHC service area	−0.01	−0.21	0.02	0.15
**Items included in composite measure of SES of CHC service area**				
High school graduation rate in zip code, %	18.4	16.2	19.2	19.8
Male unemployment rate in zip code, %	9.1	8.4	8.8	10.2
Median annual household income in zip code, $	49,231	49,005	49,948	48,755
Households below federal poverty level, %	20.0	19.6	20.0	20.3
Households with children headed by a woman, %	12.9	12.9	12.8	13.0
Households receiving public assistance, %	4.4	3.9	4.4	4.8

Abbreviations: ACO, accountable care organization; FTE, full-time equivalent; SES, socioeconomic status.

a Source: A web-based survey completed by CHC medical directors in summer 2017 about the strategies they adopted to support primary and specialty care integration and to improve CHC/specialist communication with specialists outside CHCs. CHCs were surveyed in the following states: California, Colorado, District of Columbia, Illinois, Louisiana, Maine, Minnesota, New Jersey, Oregon, Utah, Vermont, Washington, and Wisconsin.

b Using a 1-factor confirmatory factor analysis, we constructed a composite measure of CHC/specialist integration to summarize the breadth of strategies used by CHCs to achieve greater integration with specialty care providers. We categorized CHCs as having low, medium, and high levels of integration by dividing CHCs into tertiles based on the composite.

c The composite measure of SES has a mean of 0 and standard deviaion of 1. A value <0 denotes a service area with an SES level below the mean.

CHCs reported using various strategies to support the integration of primary and specialty care (Table 2). Most commonly, 80.5% of CHCs reported that on-site specialists provided any care during the previous 6 months. CHCs also frequently reported participating in quality improvement projects with specialty practices or participating in joint health promotion activities (71.2%) and having established agreements with specialists about the types of referrals specialists will accept or information the health center will provide when making a referral (70.2%). Less common strategies for CHC/specialist integration reported by CHCs include using e-consults (43.7%) and telemedicine (29.3%) and the ability to read the EHRs of specialists in real-time (24.7%). CHCs in the highest tertile of CHC/specialist integration were consistently more likely to report engaging in strategies supporting CHC/specialist integration than CHCs in the lowest tertile of integration (Table 2). For example, CHCs in the highest tertile were more likely to report making appointments on behalf of patients (67.1% vs 47.9%) and report the ability to read specialists’ EHRs in real time (38.4% vs 15.5%).

**Table 2:** Strategies Used by Community Health Centers (CHCs) (N = 215) to Support the Integration of CHCs and Specialty Care Providers, 2017^a^

Strategy	All CHCs, %	By Tertile of Integration, %^b^		
		Lowest	Middle	Highest
**Improving referrals**				
Establish agreements with specialists about the types of referrals specialists will accept or information the health center will provide when making a referral	70.2	42.3	74.7	93.2
Make appointments with specialists on behalf of CHC patients	58.6	47.9	60.6	67.1
Participate in electronic consults (e-consults) with specialists	43.7	29.6	42.3	58.9
Remind CHC patients of upcoming appointments with specialists	36.3	19.7	32.4	56.2
**Aligning goals with specialists**				
Participate in quality improvement projects or health promotion initiatives with specialists^c^	71.2	18.3	95.4	99.2
**Exchanging information**				
No electronic exchange of patient information	49.8	66.2	46.5	37.0
Send data electronically to specialist (without real-time EHR access)	25.6	18.3	33.8	24.7
Read specialists’ EHRs in real time	24.7	15.5	19.7	38.4
**Improving access**				
Specialists on-site at the health center provided any care during the past 6 months	80.5	70.4	83.1	87.7
Few affiliations with local hospitals or health systems among CHC physicians impact CHC’s ability to obtain timely specialty care for patients	41.3	26.2	25.7	18.3
Participate in telemedicine (excluding e-consults) with specialists	29.3	28.2	19.7	39.7

Abbreviations: EHR, electronic health record.

a Source: A web-based survey completed by CHC medical directors during summer 2017 about the strategies they adopted to support primary and specialty care integration and to improve CHC/specialist communication with specialists outside CHCs. CHCs were surveyed in the following states: California, Colorado, District of Columbia, Illinois, Louisiana, Maine, Minnesota, New Jersey, Oregon, Utah, Vermont, Washington, and Wisconsin.

b Using a 1-factor confirmatory factor analysis, we constructed a composite measure of CHC/specialist integration to summarize the breadth of strategies used by CHCs to achieve greater integration with specialty care providers. We categorized CHCs as having low, medium, and high levels of integration by dividing CHCs into tertiles based on the composite.

c Participation in quality improvement initiatives and participation in health promotion initiatives with specialists were combined into a single variable because the 2 items were highly correlated.

Higher levels of CHC/specialty integration were associated with higher rates of cancer screening in analyses that adjusted for possible confounders (Table 3). For example, the highest tertile of CHC/specialty integration was associated with a 5.6 percentage-point higher colorectal cancer screening rate (*P *= .047) and a 6.8 percentage-point higher cervical cancer screening rate (*P *= .01) compared with the lowest tertile. Similarly, the middle tertile of CHC/specialty integration was associated with a 5.4 percentage-point higher colorectal cancer screening rate (*P *= .04) and a 7.0 percentage-point higher cervical cancer screening rate (*P *= .008) compared with the lowest tertile. We found no significant differences between CHCs in the middle tertile and CHCs in the highest tertile of integration. Results of our falsification tests found no significant association between CHC/specialty integration and healthy weight screening for adults and tobacco screening and cessation intervention for adults, outcomes we hypothesized would not be affected by CHC/specialty integration.

**Table 3:** Association Between Integration of Community Health Centers (CHCs) and Specialty Care Providers and Screening for Colorectal and Cervical Cancers, 2017^a^

Factor	Coefficient (SE) [*P* Value]	
	Association With Rate of Colorectal Cancer Screening	Association With Rate of Cervical Cancer Screening
**Tertile of integration^b^**		
Lowest	Reference	Reference
Middle	5.35 (2.62) [.04]	6.99 (2.60) [.008]
Highest	5.59 (2.79) [.047]	6.83 (2.69) [.01]
**CHC characteristics**		
Number of service sites	0.04 (0.11) [.70]	0.18 (0.10) [.06]
Racial/ethnic minority patients, %	0.09 (0.08) [.24]	0.23 (0.07) [.001]
Uninsured patients, %	−0.35 (0.12) [.005]	−0.21 (0.12) [.09]
Medicaid patients, %	−0.33 (0.10) [.002]	−0.11 (0.10) [.30]
Primary care FTE per 10,000 patients	0.19 (0.19) [.31]	0.49 (0.21) [.02]
Enabling-service FTE per 10,000 patients	−0.12 (0.08) [.12]	−0.12 (0.09) [.21]
CHC has a chaotic environment	−2.99 (3.04) [.33]	−1.63 (2.78) [.56]
Physician turnover affects quality	3.19 (2.30) [.17]	−1.53 (2.18) [.48]
**Regional characteristics**		
Rural	−1.68 (3.53) [.63]	−2.29 (3.58) [.52]
Socioeconomic status of CHC service area	−0.37 (1.58) [.82]	−2.75 (1.42) [.05]
State-expanded Medicaid	−0.67 (4.27) [.88]	−6.89 (4.16) [.10]
State has Medicaid ACO	−0.63 (2.52) [.80]	2.16 (2.46) [.38]
**Intercept**	58.4 (8.5) [<.001]	48.3 (8.4) [<.001]

Abbreviations: ACO, accountable care organization; FTE, full-time equivalent.

a Source: A web-based survey completed by CHC medical directors (N = 215) during summer 2017 about the strategies they adopted to support primary and specialty care integration and to improve CHC/specialist communication with specialists outside CHCs. CHCs were surveyed in the following states: California, Colorado, District of Columbia, Illinois, Louisiana, Maine, Minnesota, New Jersey, Oregon, Utah, Vermont, Washington, and Wisconsin.

b Using a 1-factor confirmatory factor analysis, we constructed a composite measure of CHC/specialist integration to summarize the breadth of strategies used by CHCs to achieve greater integration with specialty care providers. We categorized CHCs as having low, medium, and high levels of integration by dividing CHCs into tertiles based on the composite.

We also found an association between CHC/specialist integration and the 4 measures of CHC/specialist communication (Figure). CHCs in the highest tertile of integration were estimated to have better rates of CHC/specialist communication, adjusting for a range of CHC characteristics, when compared with CHCs in the lowest tertile of integration. For example, 64.6% of CHCs in the highest tertile of integration reported knowing the outcome of a specialist visit compared with 41.7% of CHCs in the lowest tertile of integration (*P* = .007). The largest differences between CHCs in the highest and lowest tertiles were in the extent to which CHCs reported receiving clear recommendations on follow-up and care management after a specialist visit (26.2 percentage-point difference; *P* < .001) and the extent to which CHCs knew that a specialty visits happened (24.7 percentage-point difference, *P* = .004). The only significant difference observed between the middle and lowest tertiles was for the extent to which CHCs reported receiving clear recommendations on follow-up and care management after a specialist visit (15.6 percentage-point difference, *P* = .04).

**Figure:**
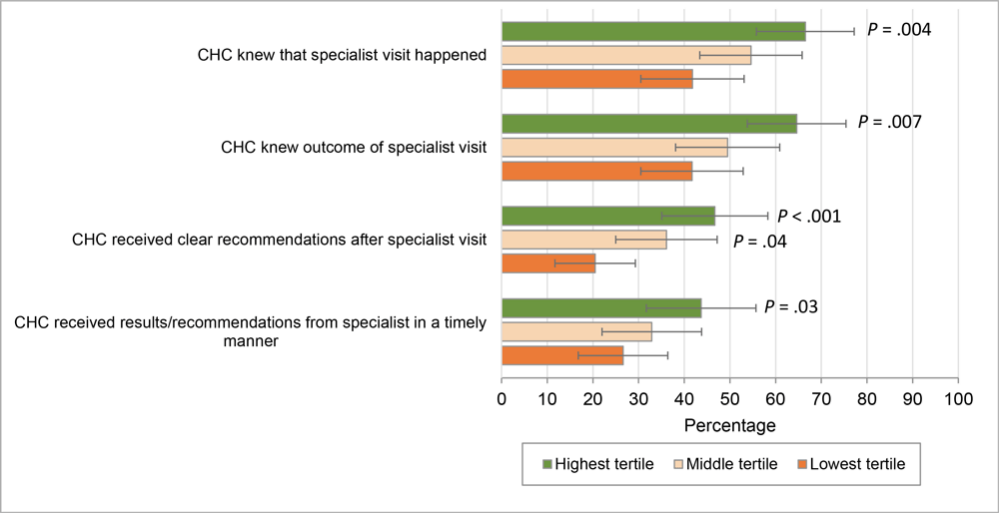
Predicted probabilities of measures of CHC/specialist communication by tertile of CHC/specialist integration. We used 4 items related to CHC/specialist communication as dependent variables to indicate whether the CHC “often” or “always” 1) knew that a specialist visit happened, 2) knew the outcome of a specialty visit, 3) received clear recommendations on follow-up and care management after the specialist visit, and 4) received results or recommendations from the specialist in a timely manner. Each item was dichotomized according to the empirical distribution of responses (reference group combined responses of “never,” “rarely,” and “sometimes”). *P* values are for comparisons with the lowest tertile. Abbreviation: CHC, community health clinic.

**Table:** 

Item	Lowest Tertile of Integration, Predicted Probability, % (95% CI)	Middle Tertile of Integration, Predicted Probability, % (95% CI) [*P *Value]^a^	Highest Tertile of Integration, Predicted Probability, % (95% CI) [*P* Value]^a^
CHC received results/recommendations from specialist in a timely manner	26.6 (16.9–36.4)	32.9 (22.1–43.8) [.40]	43.7 (31.8–55.7) [.03]
CHC received clear recommendations after specialist visit	20.5 (11.7–29.3)	36.1 (25.1–47.2) [.04]	46.7 (35.0–58.3) [<.001]
CHC knew outcome of specialist visit	41.7 (30.5–52.9)	49.5 (38.1–60.9) [.34]	64.6 (53.9–75.4) [.007]
CHC knew that specialist visit happened	41.8 (30.5–53.1)	54.6 (43.3–65.8) [.13]	66.5 (55.8–77.2) [.004]

## Discussion

In this multistate, cross-sectional study of CHCs, we found that CHCs most strongly integrated with specialists had higher rates of screening for colorectal and cervical cancers and were more likely to report successful CHC/specialist communication than the least integrated CHCs. The integration of specialists and CHCs is recommended as a strategy for increasing cancer screening in CHCs (8), and this study provides additional evidence in support of this approach. Stronger integration likely reduces barriers related to obtaining referrals for patients needing invasive testing or follow-up care, which has previously been reported by CHCs as a barrier to cancer screening (7). Additionally, more strongly integrated CHCs may have implemented effective strategies to strengthen referral processes, such as ensuring that specialists have all needed tests and information at the time of the patient’s visit, limiting referrals to only those that are necessary, and reducing no-shows through patient reminders (20). Conversely, at CHCs that are poorly integrated with specialists, patients may struggle to navigate multiple providers, miss appointments, and fail to complete screenings once they are referred to specialists, a challenge previously reported by CHCs in Washington state (9).

Along with higher rates of cancer screening, we found that stronger CHC/specialist integration was associated with better communication between CHCs and specialists. Information exchange between CHCs and specialists is essential for successful cancer screening programs. For example, poorly integrated CHCs might not receive the results of cancer screenings conducted outside their clinic, and thus have no record of completed screenings, which could lead to undercounting of screenings or duplicative screenings. O’Malley and Reschovsky reported a disconnect between information shared between primary care providers and specialists: more specialists reported sending results to primary care providers than primary care providers reported receiving such results, and vice versa (21). Real-time access to specialists’ electronic health records can address this challenge by allowing CHC staff to obtain test results, notes, and follow-up care recommendations when needed rather than relying on specialists to send this information; real-time access to specialist records was reported by 38% of the CHCs most integrated with specialists in our sample compared with 16% of the CHCs least integrated with specialists. Importantly, these efforts require all levels of staff. Director-level staff members can implement system-level strategies to facilitate CHC and specialist relationships, while these relationships are maintained by the coordination activities of staff members such as patient navigators.

Additionally, we found that nearly three-quarters of CHCs participated in quality improvement projects or health promotion initiatives with specialists in their communities. Engaging in such activities can help solidify local communities of practice. Taplin and colleagues found that screening rates for colorectal and cervical cancer increased among 4 CHCs that had established communities of practice with specialists and other community stakeholders and engaged in a regional cancer collaborative demonstration project (22). Additionally, building formal partnerships with local community hospitals and teaching hospitals may help promote access to specialty care for CHC patients (23). Furthermore, collaborations between CHCs and specialists are now supported by the HRSA’s Health Center Program statute, which outlines the requirements CHCs must meet to receive federal funding. In August 2018, HRSA updated the Health Center Program statute to explicitly require CHCs “to make every reasonable effort” to collaborate with nearby specialists (24). HRSA should monitor these integration efforts and support implementation research that can identify the most effective integration strategies, facilitate the development of quality measures that can assess uptake of these strategies, and, overall, accelerate the delivery of effective approaches.

Despite the efforts by CHCs, barriers to stronger CHC/specialist integration remain, particularly CHCs’ patient mix, which is overwhelmingly Medicaid (53.2%) or uninsured (20.4%). CHCs have historically reported difficulty in obtaining specialist appointments and procedures for their uninsured and Medicaid patients (12,25,26). Establishing referral agreements with specialists is a recommended strategy for improving referral success, and a strategy reported by more than 70% of CHCs in this study, but establishing these agreements can be challenging if specialists in the community are unwilling to accept referrals for uninsured patients (27). Furthermore, particularly for rural CHCs, there may be few specialists in close proximity with whom to coordinate (28). Additionally, smaller CHCs may have fewer resources and staff to devote to pursuing and maintaining these relationships. Thus, various strategies are likely needed to increase cancer screening for CHC patients.

This study has several limitations. In this cross-sectional study, we cannot determine whether stronger integration promotes cancer screening, or whether better-performing CHCs are more likely to pursue integrated care strategies. Although we developed a set of integration strategies used by CHCs based on a review of the literature and input from experts, other strategies may exist that we did not capture. Additionally, as with all survey research, survey responses may be affected by social desirability bias if respondents answered the items in a way that they thought would be viewed favorably rather than reflecting actual care in their CHC. Furthermore, although we conducted cognitive testing in an attempt to ensure survey items elicited the intended information, we cannot be certain that all items were interpreted as intended. Although the model fit statistics estimated for our integration measure slightly exceeded standard recommendations (ie, comparative fit index >0.90 and root mean square error of approximation <0.10), suggesting that the included items might support more than 1 underlying factor, we considered the interpretability of a single-factor model to be more important than meeting goodness-of-fit criteria (29,30). Finally, although we studied a geographically diverse sample of states and respondents varied in extent of integration and rates of screening, our convenience sample of states is not nationally representative, which may limit the generalizability of these findings nationally. As a comparison, average rates of screening for colorectal cancer (CHCs studied, 43.1% vs 42.0% nationally) and cervical cancer (54.5% CHCs studied vs 55.7% nationally) were similar for CHCs studied and CHCs nationally.

We found that CHCs that were strongly integrated with specialists reported higher cancer screening rates and better communication with specialists than the least integrated CHCs. This finding is important given the low rates of colorectal and cervical cancer screening in CHCs and, because CHCs serve a racially/ethnically diverse and economically disadvantaged patient population, increasing rates of cancer screening in CHCs can help to reduce overall inequities in cancer screening. CHCs are using various strategies to integrate care with specialists and to meet the needs of their patients, and more strategies are likely to be developed and adopted in response to HRSA’s policy change that further emphasizes CHCs’ need to integrate care with specialists. Further research is needed to better understand the challenges faced by CHCs that are weakly integrated with specialists and to determine how to help them realize the potential benefits of greater integration for the quality of care for their patients.

## Appendix Survey Items Used to Examine Integration of Community Health Centers With Specialists

**Table:**

Survey items	Response Options	Variable Construction	Included in Integration Measure
**CHC/specialist communication**			
1, In the past 6 months, for your health center’s patients who needed services from specialists outside of your health center, how frequently did clinicians or staff in your health center . . .	Never, rarely, sometimes, often, always	Dichotomous variable (often/always vs never/rarely/sometimes)	
A) Know that a visit to a specialist happened			
B) Know the outcome of the visit			
C) Receive clear follow-up or care management recommendations when needed following the specialist visit			
D) Receive results or recommendations from the specialist in a timely manner			
**CHCs’ integration with specialists**			
2. Please indicate the number of patients for whom your health center sought to obtain specialty care over the past 6 months	No patients, a few patients, some patients, most patients, all patients	Dichotomous variable (no patients vs a few patients/some patients/most patients/all patients)	
A) Specialists outside of your health center through electronic consults (e-consults)?			X
B) Specialists outside of your health center through telemedicine applications other than e-consults?			X
3. During the past 2 years, how often has your health center participated in quality improvement projects with specialists outside of your health center?	Never, rarely, sometimes, often, always	Dichotomous variable (often/always vs never/rarely/sometimes)	X
4. During the past 2 years, how often has your health center participated in health promotion initiatives (eg, hypertension awareness) with specialists outside of your health center?	Never, rarely, sometimes, often, always	Dichotomous variable (often/always vs never/rarely/sometimes)	X
5. Over the past 6 months, please indicate the number of patients for whom your health center sought to obtain specialty care via specialists on-site at your health center?	No patients, a few patients, some patients, most patients, all patients	Dichotomous variable (no patients vs a few patients/some patients/most patients/all patients)	X
6. In the past 6 months, for your health center’s patients who needed services from specialists outside of your health center . . .	Never, rarely, sometimes, often, always	Dichotomous variable (often/always vs never/rarely/sometimes)	
A) How frequently did clinicians or staff in your health center make appointments with specialists on behalf of your health center’s patients?			X
B) How frequently did clinicians or staff in your health center remind your health center’s patients of upcoming appointments with these specialists?			X
7. Please indicate how often the following factors impact your health center’s ability to obtain timely specialty care for its patients: Few affiliations with local hospitals or health systems among your health center’s physicians?	Never, rarely, sometimes, often, always, not applicable	Dichotomous variable (never/rarely vs often/always/sometimes)	X
8A. Does your health center have written or verbal agreements with specialists about either the types of referrals specialists will accept or information your health center will provide when making a referral?	Yes, no	These 3 items were combined into a categorical variable indicating 1) yes to A or B; 2) no to A and B, but yes to C, or (3) no to A, B, and C.	X
8B. Does your health center have written or verbal agreements with specialists about the type of information specialists will provide to the health center following the visit?	Yes, No		
9. Has your health center sought to establish referral agreements with one or more specialists?	Yes, No		
A) If the CHC answered yes to 8A above, then does the agreement mention the types of patients to be referred to the specialist (eg, patients with specific symptoms or conditions)?	Never, rarely, sometimes, often, always	These 5 items were combined into a count of the number of items that were "always" included in agreements with specialists (range, 0–5)	X
B) If the CHC answered yes to 8A above, then does the agreement mention any testing to be conducted prior to a referral to the specialist?			
C) If the CHC answered yes to 8A above, then does the agreement mention the information to be provided at the time of a referral to the specialist (eg, test results, patient’s medical record or clinical notes)?			
D) If the CHC answered yes to 8B above, then does the agreement mention that the specialist send a visit summary to the health center following the specialist visit?			
E) If the CHC answered yes to 8B above, then does the agreement mention the time frame by which specialists should send information to the health center following the specialist visit?			
10. Excluding faxed or scanned documents, how often does your health center . . .			
A) Send health information electronically to specialists outside your health center?	Never, rarely, sometimes, often, always	Dichotomous variable (often/always vs never/rarely/sometimes)	X^a^
B) Receive health information electronically from specialists outside your health center?	Never, rarely, sometimes, often, always	Dichotomous variable (often/always vs never/rarely/sometimes)	
11. How often are your staff able to read, in real time, the medical records of the specialty practices to which you refer your patients?	Never, rarely, sometimes, often, always	Dichotomous variable (often/always vs never/rarely/sometimes)	X
**Characteristics of CHCs**			
12. Please indicate how often the following factors impact your health center’s ability to care for its patients.	Never, rarely, sometimes, often, always, not applicable	Dichotomous variable (often/always vs never/rarely/sometimes)	
A) Chaotic environment within your health center			
B) Physician turnover			

^a^ Combined with item below using an “OR” statement.
